# The Effect of a *Tribulus*-Based Formulation in Alleviating Cholinergic System Impairment and Scopolamine-Induced Memory Loss in Zebrafish (*Danio rerio*): Insights from Molecular Docking and In Vitro/In Vivo Approaches

**DOI:** 10.3390/ph17020200

**Published:** 2024-02-02

**Authors:** Salwa Bouabdallah, Ion Brinza, Razvan Stefan Boiangiu, Mona H. Ibrahim, Iasmina Honceriu, Amna Al-Maktoum, Oana Cioanca, Monica Hancianu, Amr Amin, Mossadok Ben-Attia, Lucian Hritcu

**Affiliations:** 1 Laboratoire de Biosurveillance de l’Environnement (LR01/ES14), Faculté des Sciences de Bizerte, Université de Carthage, Zarzouna 7021, Tunisia; mossadok.benattia@fsb.ucar.tn; 2 Department of Biology, Faculty of Biology, Alexandru Ioan Cuza University of Iasi, 700506 Iasi, Romaniahritcu@uaic.ro (L.H.); 3 Department of Pharmaceutical Medicinal Chemistry and Drug Design, Faculty of Pharmacy (Girls), Al-Azha University, Cairo 11884, Egypt; 4 Biology Department, College of Science, United Arab Emirates University, Al Ain 15551, United Arab Emirates; 5 Faculty of Pharmacy, “Grigore T. Popa” University of Medicine and Pharmacy, 16 University Street, 700115 Iasi, Romania; 6 College of Medicine, University of Sharjah, Sharjah P.O. Box 27272, United Arab Emirates

**Keywords:** Alzheimer’s disease, Ayurveda, neuroprotection, neurobiology, NOR, nutraceuticals, Y-maze

## Abstract

*Tribulus terrestris* L. (Tt) has been recently gaining attention for its pharmacological value, including its neuroprotective activities. In this study, we explore the neuroprotective effects of a *Tribulus terrestris* extract in a zebrafish (*Danio rerio*) model of scopolamine (SCOP)-induced memory impairment and brain oxidative stress. SCOP, an anticholinergic drug, was employed to replicate fundamental aspects of Alzheimer’s disease (AD) in animal models. The fish were treated with ethanolic leaf extract (ELE) from Tt (1, 3, and 6 mg/L) for 15 days. SCOP (100 µM) was administered 30 min before behavioral tests were conducted. Molecular interactions of the major compounds identified via UPLC-PDA/MS in Tt fractions with the active site of acetylcholinesterase (AChE) were explored via molecular docking analyses. Terrestrosin C, protodioscin, rutin, and saponin C exhibited the most stable binding. The spatial memory performance was assessed using the Y-maze test, and memory recognition was examined using a novel object recognition (NOR) test. Tt extract treatment reversed the altered locomotion patterns that were caused by SCOP administration. Biochemical analyses also verified Tt’s role in inhibiting AChE, improving antioxidant enzyme activities, and reducing oxidative stress markers. The present findings pave the way for future application of Tt as a natural alternative to treat cognitive disorders.

## 1. Introduction

With the rise in life expectancies worldwide, age-related diseases have become a global health priority. The number of individuals affected by dementia is anticipated to rise to 152 million by 2050 [1]. Alzheimer’s disease (AD) is the major cause of dementia and accounts for 60–75% of dementia cases [2]. It is characterized by its neurodegenerative nature and manifests as memory-related cognitive impairment and functional decline. The development of AD is primarily governed by genetic factors, but its onset can be linked to exposure to environmental pollutants such as heavy metals and pesticides [3]. One of the defining features of AD is a reduction in acetylcholine (ACh) in the brain. This cholinergic hypothesis has been targeted in the treatment of AD. Approved drugs such as galantamine (GAL), rivastigmine hydrogen tartrate, huperzine-A, and donepezil enhance cholinergic neurotransmission by inhibiting acetylcholinesterase (AChE) activity [4,5]. These medications offer symptomatic relief but cannot reverse the progression of AD and can lead to various adverse side effects [6,7].

The discovery of new drugs from remedies used in traditional medicine has been an appealing approach that is continuously progressing. Previous research has directed attention toward developing therapies from natural products that could help in preventing and managing sustained neurodegeneration in AD [8]. For instance, phytochemicals such as curcumin and resveratrol have been shown to exert neuroprotective activities against key AD hallmarks, including amyloid-beta accumulation, oxidative stress, and neuroinflammation [9]. Some of these compounds have exhibited great efficacy accompanied by low toxicity [10,11]. Therefore, using plants in the pursuit of novel AD treatments is an appropriate strategy.

*Tribulus terrestris* (Tt) is a popular medicinal herb that is commonly used in Chinese, Indian, and Bulgarian folk medicine [12]. It is traditionally used for its aphrodisiac, diuretic, anticonvulsant, and antihypertensive properties [13]. These traditional uses of Tt have been substantiated by modern research [14,15,16], attracting scientific attention with numerous studies that highlight its pharmacological potential. This includes its anticancer, antioxidant, antileishmanial, antidiabetic, immunomodulatory, and anti-inflammatory effects [17,18,19,20,21]. Phytochemical analysis of Tt has revealed its abundance of a wide range of structurally diverse compounds, namely steroidal saponins [22], flavonoids, tannins, glycosides, phytosterols, and terpenoids [23]. Steroidal saponins and flavonoids are the predominant metabolites that give Tt its pharmacological activities. Over a hundred steroidal saponins have been identified from Tt, including protodioscin, diosgenin, dioscin, gracillin, and trillin [23,24]. Recently, these steroidal saponins have been shown to possess neuroprotective effects against AD [25,26,27].

Zebrafish (*Danio rerio*) have gained prominence as an effective tool for exploring the impacts of various compounds in neurobiology [28,29,30,31]. They offer a great advantage in biomedical research due to their genetic resemblance to humans and simplicity of maintenance [31]. Both larval and adult zebrafish have been widely employed for investigating neurological processes and impairments [32]. The scientific literature substantiates the benefit of using zebrafish to investigate the cholinergic hypothesis of AD [33]. SCOP is a cholinergic receptor antagonist that has been widely used to construct animal models of cognitive deficits associated with learning and memory. It induces central features of AD including amyloid-beta accumulation and cholinergic dysfunction [34,35].

The current research focuses on investigating the potential of ethanolic leaf extracts (ELE) from Tt to prevent scopolamine (SCOP)-induced cognitive dysfunction in zebrafish. The study also aims to understand how Tt impacts the antioxidant capacity of the zebrafish brain in a model induced with SCOP, a compound known for inducing cognitive impairment comparable to Alzheimer’s disease (AD). Additionally, our investigation incorporates a docking study to assess the interactions between the substances and the active site of acetylcholine esterase using computational techniques. This approach provides a predictive understanding of the structure of the complex formed between a ligand (potentially from ELE) and the enzyme. The utilization of molecular docking has gained significant prominence as a pivotal tool in the field of drug discovery.

## 2. Results

### 2.1. Metabolite Profiling of Tribuls Terrestris Extract

**Figure 1:**
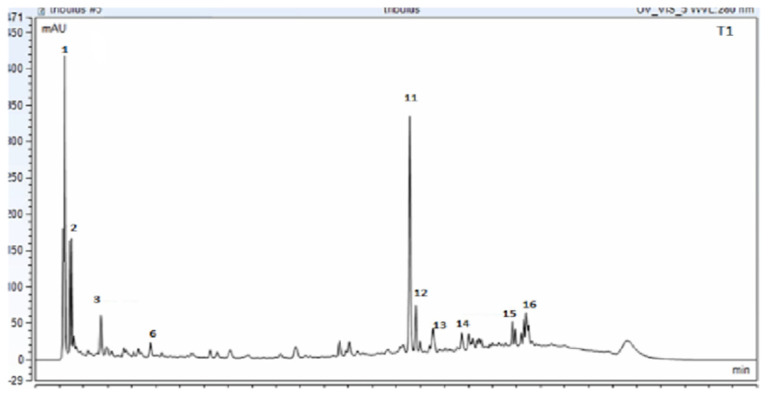
UPLC_PDA chromatogram for *Tribulus terrestris* sample (ELE: ethanolic leaf extract) at 280 nm. The confirmation of the indicated compounds was verified by MS technique (Peak number identity is displayed in Table 1).

The chromatogram results indicated the presence of several compounds in the sample, as shown in Figure 1 Tt extract was analyzed via UPLC-PDA to determine its chemical profile. The results revealed the presence of various flavonoids and saponins, including epigallocatechin, apigetrin, rutin, quercetin, luteoline, cynaroside, caffeic acid, trillin, hecogenin, terreside B, trillarin, protodioscin, and saponin C (Figure 1).

The identification of the compounds was further confirmed using the UPLC-EIS/MS method, which was based on reviewing the literature and the fragmentation patterns that were compared to similar compounds in the online database using Compound Discover 3.2 software for evaluation in both targeted and untargeted modules. The peaks with a rating of at least 7.4 were considered and verified against the Thermo *m*/*z* Vault and ChemSpider databases. In this regard, metabolite profiling of Tt has led to the tentative identification (Table 1) of a wide array of metabolites represented by phenolic acids, flavonoids, and saponins. The molecular mass ion peaks at *m*/*z* 180.04225 and 196.05823, corresponding to the suggested molecular formulas C_9_H_8_O_4_ and C_9_H_8_O_3_ [M+H]^−^, fit caffeic acid and hydroxycinnamic acid. The molecular ion mass peaks at *m*/*z* 285.0409, 301.0353, 356.07451, and 609.1482 [M+H]^−^, respectively, for the predicted molecular formulas C_15_H_10_O_7_, C_15_H_10_O_6_, C_15_H_14_O_7_, and C_27_H_30_O_16_ gave hits for kaempeferol, quercetin, epigallocatechin, and rutin [M+H]^−^. The molecular ion mass peaks at *m*/*z* 913.48023, 915.4590, 738.05805, and 1049.2 [M+H]^−,^ respectively, for the anticipated molecular formulas C_51_H_84_O_12_, C_45_H_12_O_15_, C_39_H_62_O_13_, and C_51_H_84_O_22_ gave hits for saponin C, terrostrosin C, trillarin, and protodioscin (Table 1).

### 2.2. Molecular Docking Study

AChE is a crucial enzyme that inhibits neurotransmission by hydrolyzing ACh in the synapse. AChE is predominantly found at neuromuscular junctions and cholinergic synapses in the central nervous system (CNS), where it promotes the hydrolysis of ACh into choline and acetate, thereby terminating ACh-mediated synaptic transmission with high catalytic efficiency [36]. Three core-binding regions make up the structure of AChE: the peripheral aromatic site (PAS), the catalytic active site (CAS), and the gorge. The catalytic triad of Ser203, His447, and Glu334 is present on the CAS-containing gorge bottom. Moreover, the middle gorge connects CAS and PAS and extends beyond the enzyme [36]. The majority of the inner surface is composed of aromatic amino acids (Phe295, Phe338, and Tyr337). The PAS at the gorge entrance is predominantly composed of five residues: Asp74, Tyr72, Tyr124, Tyr341, and Trp286 [37]. The docked scores of 15 compounds against AChE ranged from −11.22 to −24.68 kcal/mol, compared to −14.62 kcal/mol for donepezil (co-crystal ligand) (Table 2 and Table S1). With docking energies lower than −24 kcal/mol, terrestrosin C, protodioscin, rutin, and saponin C were the most stable docked compounds. The order of highest docking scores was as follows: rutin > saponin C > protodioscin > terrestrosin C > trillarin > epigallocatechin > terreside B > cynaroside > disogluside, apigetrin > kaempferol > quercetin > hecogenin > luteoline > donepezil > caffeic acid.

### 2.3. Effect of Tt Extract on Locomotor Activity

**Figure 2:**
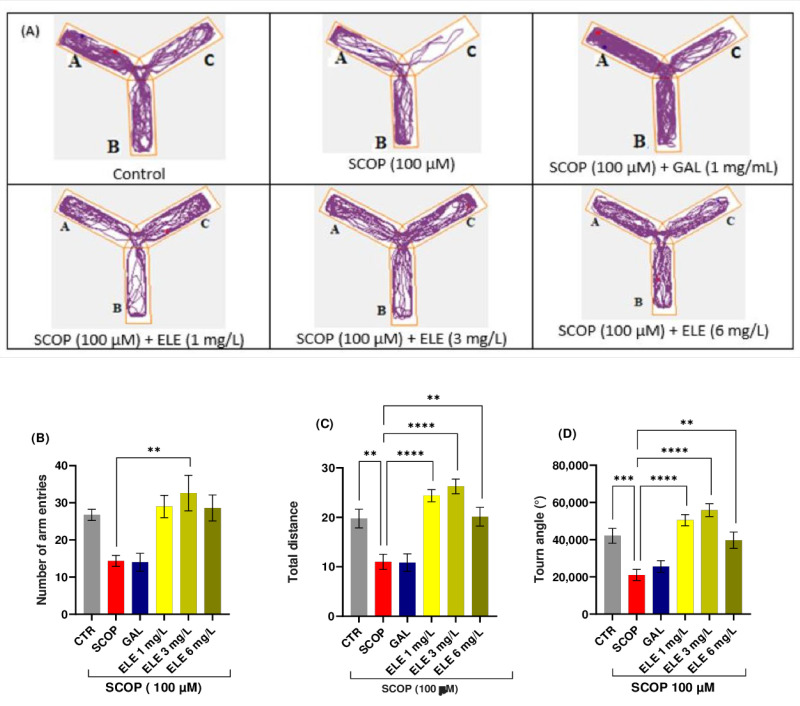
The effects of *Tribulus terrestris* (ELE, 1, 3, and 6 mg/L) administration in SCOP-treated zebrafish on spatial memory and locomotor activity assessed within the Y-maze task. (**A**) The tracking pattern of locomotion exhibited by zebrafish during the second phase of the Y-maze task based on their respective experimental group allocations. The arms of the maze were denoted with A (start arm), B (another arm), and C (novel arm). (**B**) Number of arm entries. (**C**) Total distance (m). (**D**) Turn angle (°). Data are represented by means ± S.E.M. (*n* = 8). Galantamine (GAL, 1 mg/L) was used as a reference positive drug. For Tukey’s post hoc analyses_ ** *p* < 0.01, *** *p* < 0.001, and **** *p* < 0.0001. The starting point of the zebrafish’s path is denoted by the blue dot •, while the red dot • signifies the endpoint of the fish’s trajectory.

Digital videos were recorded to observe the movement of zebrafish in water, vertically or horizontally, over a certain period of time. A trajectory drawn in the specialized software package was used to automatically track the swimming style of the zebrafish. Representative swimming behavioral parameters were analyzed. This included the means of total moving distance, velocity, or turning angle [38,39,40]. A basic approach to analyzing zebrafish movement involves counting the total number of lines crossed by the fish. The enhancement in memory observed in the Y-maze test can be attributed to locomotor activity, which is assessed by the number of entries into the maze arms. The groups pretreated with SCOP exhibited significant variations, indicated by *p* < 0.05. As depicted in Figure 2, the control group exhibited regular swimming behavior within the Y-maze tank. However, zebrafish subjected to SCOP treatment displayed an altered locomotion tracking style that was reversed by ELE administrations; *p* < 0.005 for 3 mg/L and *p* < 0.05 for both 1 and 6 mg/L. Moreover, a non-significant improvement in locomotion was observed following GAL treatment (Figure 2).

### 2.4. Effect of Tt Extract on Spatial Memory in Y-Maze

The Y-maze was used to evaluate spatial working memory in the amnesic fish. Figure 2 illustrates the memory impairment caused by SCOP as SCOP-treated fish spent minimal time in the novel arm. The administration of ELE demonstrated memory-enhancing effects in the amnesic fish. One-way ANOVA revealed a significant increase in spatial memory performance in groups treated with low and high doses (1, 3, and 6 mg/L) of Tt in terms of total distance traveled (F (8, 63) =11.33 (*p* < 0.0001 for 1 and 3 mg/L and *p* < 0.005 for 6 mg/L), time spent in the novel arm (% of total arm time F (8, 63) = 5.225, *p* < 0.01), and turn angle (one-way ANOVA F (8, 63) = 11.31, *p* < 0.001), with increased percentages compared to the group treated with SCOP alone, indicating significant effects on short-term memory. Further analysis using Tukey’s post hoc test revealed a significant difference between the control and SCOP groups (*p* < 0.0001), control and SCOP + ELE (1 mg/L) groups (*p* < 0.0001), SCOP and SCOP + ELE (3 mg/L) groups (*p* < 0.0001), and SCOP and SCOP + ELE (6 mg/L) groups (*p* < 0.001), indicating that ELE significantly improved spatial memory (Figure 2). Fish treated with ELE explored all three arms of the maze. These fish outperformed the GAL-treated group in terms of behavioral parameters. This improvement in spatial memory is consistent with previous studies investigating the effects of phytochemicals using the Y-maze test [38,39,40].

### 2.5. The Effects of Tt Extract on Recognition Memory in NOR Task

**Figure 3:**
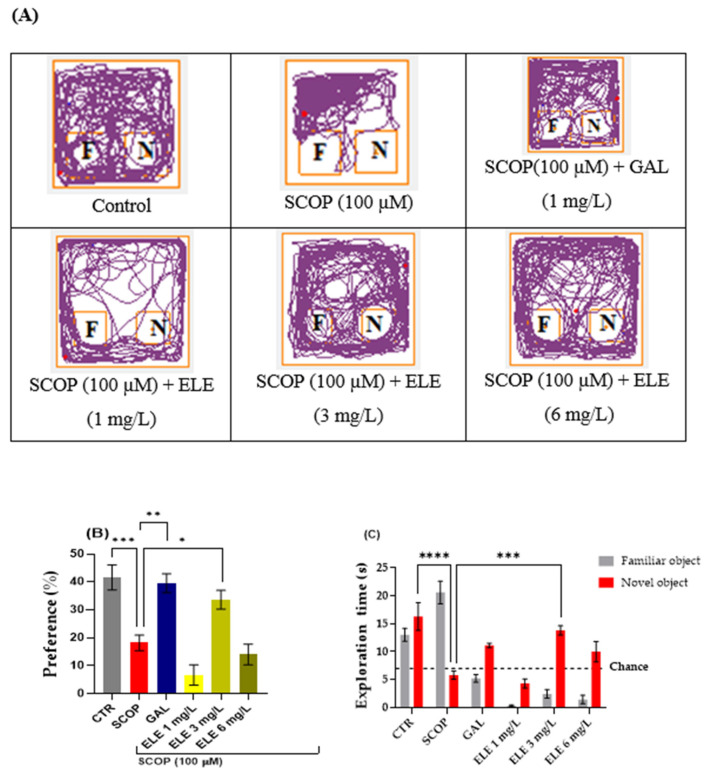
The effects of administering *Tribulus terrestris* (ELE, 1, 3, and 6 mg/L) to SCOP-treated zebrafish on recognition memory, as assessed through the object discrimination task. (**A**) The locomotor tracking patterns of the zebrafish during the object discrimination task’s testing session according to their respective experimental groups. (**B**) The duration spent in the top (S). (**C**) The measure of preference percentage was utilized as the endpoint for evaluating recognition memory. The familiar and the novel objects are denoted as F and N, respectively. The black dashed line (chance) indicates a 50% preference. Data are represented by means ± S.E.M. (*n* = 8). Galantamine (GAL, 1 mg/L) was used as a reference positive drug. For Tukey’s post hoc analyses_ * *p* < 0.05, ** *p* < 0.01, *** *p* < 0.001, and **** *p* < 0.0001. The starting point of the zebrafish’s path is denoted by the blue dot •, while the red dot • signifies the endpoint of the fish’s trajectory.

In this test, the object distinction task was performed to evaluate the recognition memory of the zebrafish. These vertebrates possess the capacity to differentiate between familiar and unfamiliar objects and remember three-dimensional geometric forms [36,41] Following the object discrimination test, we generated representative tracking plots illustrating the paths taken by the fish during the testing session, according to their respective experimental groups. The effects of the SCOP (100 µM) administration and ELE (1, 3, and 6 mg/L) treatments on NOR recognition memory and locomotion are illustrated in Figure 3 In the NOR test, the one-way ANOVA revealed a significant effect of the treatment on the preference percentages (F (8, 61) = 17.30, *p* < 0.0001) and exploratory time (F (17, 125) = 18.64, *p* < 0.0001)) (Figure 3B). The representative locomotion tracking plots disclosed changes in swimming behavior (Figure 3A). Compared to the control group, zebrafish treated with SCOP had lowered preference percentages for the novel objects (NOs), implying impaired recognition memory. Furthermore, ELE treatment (3 mg/L) increased preference percentages in SCOP-treated zebrafish (*p* < 0.05 (Figure 3), suggesting memory enhancement. As shown in Figure 3, SCOP-treated fish spent more time exploring the familiar object and less time exploring the novel object compared to fish from other groups. The one-way ANOVA indicated statistical significance (*p* < 0.05). According to Figure 3, the three doses of ELE, but especially the 3 mg/L dose, reversed the recognition memory deficits caused by SCOP administration by significantly (*p* < 0.05 and *p* < 0.01 for ELE (3 mg/L)) increasing the preference of the zebrafish for the novel object. The 3 mg/L doses of ELE demonstrated performance levels exceeding the chance level (50%), indicating a relative preference for the novel object. Therefore, the ELE improved recognition memory in zebrafish with SCOP-induced patterns of AD, as observed in the object distinction task. Saponins and flavonoids have been reported to improve recognition memory in experimental animals [42]. In one study, flavonoids rutin and naringin dose-dependently enhanced the discriminative ability of SCOP-treated animal models [43]. The present findings may be attributable to the abundance of such compounds.

### 2.6. Effects of Tt Extract on Brain AChE Activity

**Figure 4:**
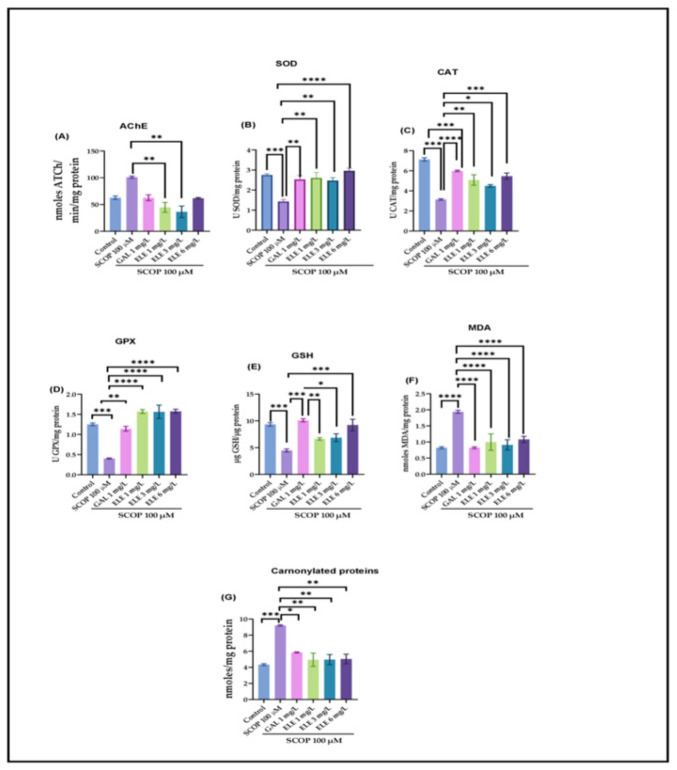
The effects of *Tribulus terrestris* (ELE, 1, 3, and 6 mg/L) administration in scopolamine (SCOP)-treated zebrafish on (**A**) AChE, (**B**) superoxide dismutase (SOD), (**C**) catalase (CAT)-specific activities, (**D**) glutathione peroxidase (GPX), (**E**) GSH, (**F**) malondialdehyde (MDA), and (**G**) carbonylated protein levels. Data are represented by means ± S.E.M. (*n* = 8). Galantamine (GAL, 1 mg/L) was used as a reference positive drug. For Tukey’s post hoc analyses_ * *p* < 0.05, ** *p* < 0.01, *** *p* < 0.001, and **** *p* < 0.0001.

To investigate the efficacy of the Tt extract treatment on the SCOP-treated zebrafish, key biochemical markers associated with cholinergic activity were measured. SCOP administration induced a significant increase in AChE activity (*p* < 0.001). This rise in AChE activity was notably decreased in the GAL group to a level comparable to that of the control group (*p* < 0.001). Similarly, Tt treatment dose-dependently lowered AChE activity (*p* < 0.005 for 3 mg/L and *p* < 0.01 for 1 mg/L), when compared to the groups treated with SCOP alone. The effects exerted by Tt surpassed those of the GAL treatment (Figure 4A). These results align with the inhibitory effects of Tt against AChE in animal models of neurodegenerative diseases [44,45] (Table S2).

### 2.7. Effects of Tt Extract on Brain Oxidative Status

The one-way ANOVA analysis indicated the considerable effects exerted by the treatment on the oxidative condition of the brain, as indicated by changes in AChE (F (8, 18) = 6.262, *p* < 0.0006) (Figure 4A), SOD (F (8, 18) = 9.002, *p* < 0.0001) (Figure 4B), CAT (F (8, 18) =15.2, *p* < 0.0001) (Figure 4C), GPX (F (8, 18) = 22.82, *p* < 0.0001) (Figure 4D), and GSH (F (8, 18) =10.60, *p* < 0.0001) (Figure 4E). Elevated levels of lipid peroxidation (MDA) (F (8, 18) = 16.02 *p* < 0.0001) (Figure 4F) and protein oxidation (carbonylated proteins) (F (8, 18) =10, *p* < 0.0001) were also observed (Figure 4G). (F (8, 18) is a notation related to statistical analysis, specifically analysis of variance (ANOVA). The “F” stands for the F-statistic, and the numbers in parentheses represent degrees of freedom. In this case, it suggests an F-test with 8 and 18 degrees of freedom. The F-statistic is used to assess whether there are significant differences among group means in an ANOVA test). Compared to the control group, the SCOP-exposed zebrafish showed a significant decrease in activities of antioxidant enzymes, such as SOD (*p* < 0.0001) (Figure 4B), GPX (*p* < 0.0001) (Figure 4D), and CAT (*p* < 0.0001) (Figure 4C), as well as elevated levels of MDA (*p* < 0.0001) (Figure 4F) and carbonylated proteins (*p* < 0.0001) (Figure 4G). ELE treatment increased antioxidant enzyme activity by decreasing SCOP-induced brain oxidative stress, as reflected in the levels of SOD (*p* < 0.005 for 1 mg/L and 3 mg/L and *p* < 0.001 for 6 mg/L) (Figure 4), GPX (*p* < 0.0001 for 1 mg/L, 3 mg/L, and 6 mg/L), CAT (*p* < 0.005 for 1 mg/L and 3 mg/L and *p* < 0.001 for 6 mg/L), and GSH (*p* < 0.001 for 6 mg/L) and a reduction in MDA (*p* < 0.0001 for 1 mg/L, 3 mg/L, and 6 mg/L) (Figure 4F) and carbonylated protein levels (*p* < 0.001 for 1 mg/L, 3 mg/L, and 6 mg/L) (Figure 4) compared to SCOP-treated zebrafish.

## 3. Discussion

Several studies have shown the presence of flavonoids in Tt, which are mainly derivatives of quercetin, kaempferol, and isorhamnetin [23]. Consistent with other published work, compounds similar to the ones presented here were identified in the leaves of Tt [12,23]. However, most published data provide general identifications without the quantifications of these compounds. The extractability is correlated with the plant material, the solvent, the geographical locality, and the preparation process [23,46]. The chemical composition shows that the investigated extract is rich in flavonoids and saponins, which are known as natural antioxidants. Their free hydroxyl groups allow interactions with cell membranes and trigger protective actions against free radicals. Additionally, rutin, quercetin, and catechin derivatives serve as barriers against reactive oxygen species and hinder oxidative enzymes such as lipoxygenase, thereby preventing cell denaturation [47].

**Figure 5:**
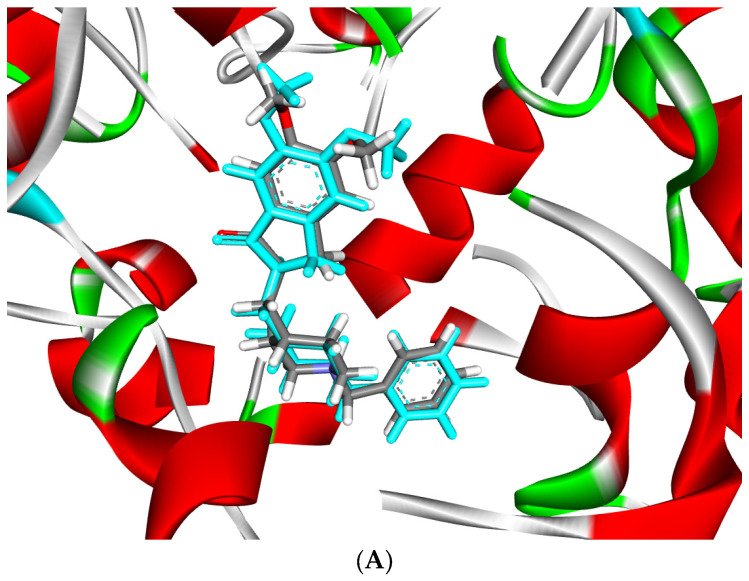

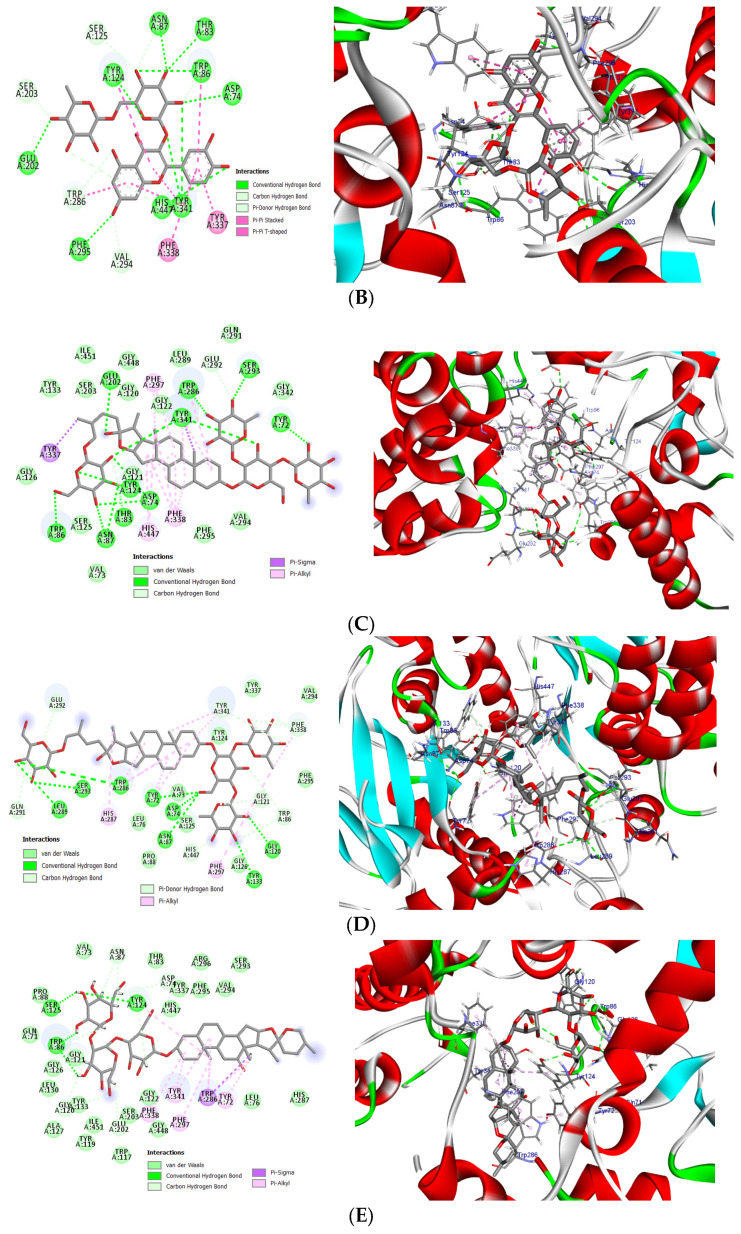
(**A**) Superposition of co-crystal ligand (donepezil) (blue color) with its docked pose (gray color) showed an appreciable RMSD of 0.47 Å. (**B**) The 2D (left) and 3D (right) binding mode of rutin in the active site of AChE enzyme. (**C**) The 2D (left) and 3D (right) binding mode of protodioscin in the active site of AChE enzyme. (**D**) The 2D (left) and 3D (right) binding mode of saponin C in the active site of AChE enzyme. (**E**) The 2D (left) and 3D (right) binding mode of terrestrosin C in the active site of AChE enzyme.

Using molecular docking, the interactions of the 15 compounds (Table 1 and Table S1) with AChE were visualized. The co-crystallized ligand donepezil was redocked into the active site of AChE (Figure 5A) to assess the accuracy of the docking. The root mean square deviation (RMSD) between the docking and original co-crystallized poses was 0.47 Å. The majority of the selected compounds exhibited favorable interactions with essential amino acid residues such as Tyr72, Asp74, Trp86, Tyr124, Ser293, Phe295, Phe297, Tyr337, Phe338, Tyr341, and Trp286. Interestingly, rutin, with a docking score of −24.68 kcal/mol, showed 11 conventional hydrogen bond interactions with the essential amino acids of the PAS and CAS regions: Tyr124 (1.64 Å), Phe295 (2.93 Å), Tyr341 (2.45 Å), Tyr341 (3.04 Å), His447 (2.85 Å), Asp74 (2.71 Å), Thr83 (2.79 Å), Asn87 (2.50 Å), Trp86 (3.12 Å), and Glu202 (3.05 Å and 3.22 Å). In addition, rutin forms six carbon–hydrogen bonds, two π-donor hydrogen bonds, and eight π-π-stacked bonds (Figure 5B, Table 3). Protodioscin displayed a binding mode similar to that of rutin and donepezil (co-crystal ligand). It formed 16 hydrogen bonds with 12 amino acids, ranging in length from 2.04 to 3.23 Å. Sugar moieties play a key role in the formation of hydrogen bonds with Trp286 (3.23 Å), Trp86 (3.20 Å), Tyr72 (2.97 Å), Asp74 (2.82 and 2.65 Å), Tyr124 (2.04 Å), Tyr341 (2.04 and 2.81 Å), Ser293 (2.15 and 2.60 Å), Asn87 (2.80 and 2.70 Å), Thr83 (2.57 and 2.78 Å), Glu202 (2.77 Å), and Glu292 (2.47 Å). The steroid portion of protodioscin contributed to 14 hydrophobic interactions through the formation of π-Sigma or π-Alkyl interactions (Figure 5C, Table 3). Additionally, saponin C (Figure 5D, Table 3) and terrestrosin C (Figure 5E, Table 3) exhibited extremely low binding energies of −24.11 and −24.63 Kcal/mol, respectively. Their steroid moieties exhibited hydrophobic interactions with Try72, Try286, Phe297, Phe338, and Try341 in the PAS region of the AChE enzyme. Furthermore, saponin C and terrestrosin C formed twelve and twenty-one hydrogen bonds, respectively.

The inhibitory effects of Tt against AChE may be due to the presence of various flavonoids and saponins, including epigallocatechin, apigetrin, rutin, quercetin, luteoline, cynaroside, t-caffeic acid, trillin, hecogenin, terreside B, trillarin, protodioscin, and saponin C (Figure 1). Additionally, in a docking study, those compounds showed good interactions with crucial amino acid residues like Tyr72, Asp74, Trp86, Tyr124, Ser293, Phe295, Phe297, Tyr337, Phe338, Tyr341, and Trp286. The most stable docked molecules were rutin, saponin C, terrestrosin C, and protodioscin, all of which had docking energies less than −24 kcal/mol. It is interesting to note that the maximum docking score of rutin was achieved at −24.68 kcal/mol. This suggests that rutin plays a significant role as an AChE inhibitor.

A recent study by Sleem et al. [44] investigated the anti-Parkinson potential of Tt using a haloperidol-induced rat pathological disorder model. This study showed that the methanolic extract of the plant possesses antioxidant potential, promoting disease amelioration by increasing the levels of endogenous antioxidant enzymes and decreasing the levels of AChE, α-synuclein, IL-1β, and TNF-α [48,49]. Other studies have indicated that astragaloside-like pentacyclic triterpene saponins can protect PC12 neuronal cell survival by activating GSK-3β and preventing mitochondrial permeability transitional pore (mPTP) opening. GSK-3β plays an important role in neural cell survival, growth, and neuronal plasticity [49,50]. Inactivation of GSK-3β to prevent mPTP opening is vital for neurological protection [50,51]. Ginsenosides, gintonin, ginseng extracts/fractions, and ginseng-containing formulas have been extensively studied in cells, demonstrating that ginseng possesses neuroprotective properties against AD. These effects are exerted via the regulation of several signaling pathways, such as the PI3K/Akt, AMPK-mTOR, and NF-κB pathways, to inhibit or ameliorate key AD features, including Aβ accumulation, neurotrophic factors, tau phosphorylation, neuroinflammation, apoptosis, and mitochondrial dysfunction at different stages of AD [5]. The blocking of the central muscarinic ACh receptor results in a disruption of learning and memory skills in both humans and animals. The anticholinergic SCOP was used as a strong amnesic agent. Various investigations have demonstrated that prolonged SCOP administration resulted in a reduction in protective antioxidant mechanisms. This occurs as a result of the suppression of nuclear factor erythroid 2-related factor 2 (Nrf2) expression and is related to decreased synaptic plasticity via the cAMP response element-binding protein (CREB)/BDNF pathway [51,52]. Moreover, SCOP-induced oxidative stress phosphorylates GSK and enhances AChE activity, which ultimately leads to apoptosis through Bax/Bcl-2 mediators [51,52]. In AD patients, the synthesis and release of ACh are delayed, while AChE is hyperactive and breaks down more ACh, obstructing cholinergic system transmission [53,54].

Models of aged animals have revealed a significant association between the development of behavioral deficits or cognitive impairments in learning and memory and spatial and temporal memory [53]. In the SCOP-induced model, Tt inhibited AChE activity, indicating positive effects on cholinergic neurotransmission. Carbonylated proteins are the products of protein oxidation [54], while CAT and SOD are considered to be two important antioxidant enzymes that counteract oxidative stress [55,56,57].

## 4. Materials and Methods

### 4.1. Extraction and Plant Material

*Tribulus terrestris* (Tt) was collected from Tunisian flora, plants grown spontaneously in the region of Elhawaria (Ain Halloufa). The samples were harvested during the mature fruit stage in September 2020. Harvested plants were identified according to Pottier-Alapetite and furthermore confirmed according to methods previously described by our botanist colleagues as mentioned in our last publication [20]. The extraction protocol was performed as previously reported by Bouabdallah et al. [20,21] with slight modifications. Fresh leaves of *T. terrestris* were dried in the shade for two weeks and then reduced to a fine powder. One hundred grams of the resulting powder was extracted using 70% *v*/*v* ethanol (reflux at 80 °C, 3 × 1000 mL × 2 h). The obtained solutions were combined and concentrated under a vacuum at 70 °C to a small volume.

### 4.2. Chemical Analysis of the Investigated Extract

#### 4.2.1. Ultrahigh-Performance Liquid Chromatography–Photodiode Array Detection (UPLC-PDA)

An UltiMate 3000 Thermo Fisher system coupled with a photodiode array (PDA) detector was used for the mass spectrometry (MS) analysis. The mobile phase included a gradient of A (acetonitrile with 0.1% phosphoric acid) and B (0.1% phosphoric acid) as follows: 0–4 min, 10–15%; the next 4 min, isocratic at 15%; 8–15 min, 30%; 15–18 min, 40%; 18–22 min, 55%; then for the next 3 min, the system returned to the initial conditions. The column used was Luna Omega 5 µ Polar C18 (100 A, 150 × 4.6 mm). The sample injection was 2 µL with a flow rate of 0.8 mL. Detection was performed at seven different band wavelengths between 220 nm and 800 nm, considering the four detection bands 245 nm, 280 nm, 330 nm, and 521 nm. These wavelengths represent the maximum absorption bands for flavonoids, phenolic acids, and anthocyanidins. Every peak was identified based on UV spectra and the available library data, and the identifications were compared to the standards. Calibration curves were established for multiple standards, including catechin, epicatechin, ellagic acid, caffeic acid, p-coumaric acid, cinnamic acid, chlorogenic acid, ferulic acid, luteolin, apigenin, kaempferol, epigallocatechin, quercetin, rutoside, quercetin-3-O-arabinoside, apigenin-7-O-glucoside, luteolin-7-O-glucoside, and ecdysone, for the quantification of the identified compounds.

#### 4.2.2. MS Liquid Chromatography (UPLC-ESI/MS)

A system comprising a Transcend TLX-1 Vanquish Flex chromatograph coupled with an Orbitrap Exploris 480 mass spectrometer was used to further assess the chemical composition of the investigated extract. The full scan evaluation was carried out on a Hypersil Gold C18 column (50 × 2.1 mm, 1.9 µm) thermostated at 40 °C. The separation of the active compounds was achieved with a gradient mixture of A (acetonitrile with 0.1% phosphoric acid) and B (0.1% phosphoric acid) over 30 min, from 7% to 93% A in B. The flow rate was 0.3 mL. Positive and negative electrospray ionization (ESI) was employed for mass spectrometric detection. Acquired spectra were recorded within a mass range of *m*/*z* 100 to 1500 at a frequency of 5 Hz, using a positive voltage of 4.5 kV, a negative voltage of 3.0 kV, and an ionization temperature of 350 °C. Aliquots of 5µL from each sample were injected automatically. Integration and detection were carried out using Compound Discoverer 3.2 software for evaluation in both targeted and untargeted modules. The peaks with a rating of at least 7.4 were considered and verified against the Thermo *m*/*z* Vault and ChemSpider databases.

### 4.3. Molecular Docking

With the assistance of the Molecular Operating Environment (MOE)-Dock 2019.09 program, a docking analysis was conducted. Using the constructor key, sketches of the selected molecules’ structures were generated. The MMFF94x force field, which is a standard feature of the MOE program, was then utilized to reduce the energy level of these compounds. Subsequently, using a conformer search, 3D conformers of these compounds were obtained. AChE was acquired from the protein data bank (PDB ID: 4EY7) [58] and was imported into MOE. Water molecules were removed, and missing hydrogens were added to generate the necessary ionization conditions of the protein structure. The location of the active site was determined using the MOE Alpha Site Finder with its default parameters. The obtained alpha spheres were used to construct the inactive site’s artificial atoms. The docking process was conducted using the “Docking” component of MOE, following the standard docking procedure. The top five poses, as ranked by the London Dispersion Grid (dG), were saved, further reducing the use of the Merck Molecular Force Field 94x (MMFF94x) within the enzyme. The Generalized-Born Volume Integral/Weighted Surface Area (GBVI/WSA) dG ranking utility was then used to assign scores to the generated postures. The poses of the compounds with the highest scores were selected. We utilized the Biovia discovery-studio 2020 visualizer to examine the representation of protein–ligand interactions in the active site of the complex [59].

### 4.4. Ethical Approval

The maintenance and treatment of the fish were carried out in accordance with the EU Commission Recommendation (2007), Directive 2010/63/EU of the European Parliament, and the Council of 22 September 2010 guidelines for accommodation, care, and protection of animals used for experimental and other scientific purposes. This protocol was approved by the Ethics Committee of the Faculty of Biology, “Alexandru Ioan Cuza” University, Iasi, with registration number 370/4.02.2022. All procedures were conducted in compliance with legal and “Animal research: reporting of in vivo experiments (ARRIVE)” guidelines while ensuring minimal zebrafish utilization.

### 4.5. Fish Husbandry

A total of 100 adult wild-type short-fin strain zebrafish were obtained from Pet Product S.R.L. (Bucharest, Romania). They included both female and male zebrafish in a 1:1 ratio, with an age range of 3 to 4 months, measuring 3–4 cm in length, and weighing between 0.3 and 0.5 g. The zebrafish were acclimated for a period of two weeks in the animal facility of the Faculty of Biology at Alexandru Ioan Cuza University of Iasi, Romania. They were housed in three 70 L aquariums and maintained within a recirculation system that provided well-ventilated and dechlorinated water at a controlled temperature (26 °C ± 2). The photoperiod was set to 14:10 h (light:dark cycle). Water quality parameters were kept at constant levels (pH = 7.5, dissolved oxygen at 7.20 mg/L, ammonium concentration < 0.004 ppm, and conductivity level of 500 µS).

### 4.6. Drug Treatment and Group Division

**Figure 6:**
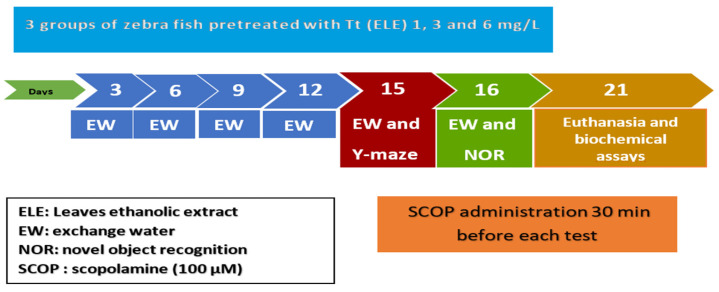
Experimental design procedure for drug administration, behavioral studies (NOR and Y-maze tests), and biochemical assays.

Zebrafish were divided into the following groups: three Tt pretreatment groups including three concentrations (ELE): 1, 3, and 6 mg/L; the control group (DMSO 1% was added via immersion); the SCOP group (SCOP, 100 µM, Sigma–Aldrich, Darmstadt, Germany); and the galantamine (GAL) group (GAL, 1 mg/L, Sigma–Aldrich, Darmstadt, Germany) serving as a positive control in the Y-maze and NOR tests. The doses of SCOP, GAL, and plant extract were selected based on previous studies [60]. ELE (1, 3, and 6 mg/L) was administered through immersion once daily in a 6 L glass for 1 h, while SCOP (100 µM) was administered 30 min preceding the behavioral tests (Figure 6).

### 4.7. Behavioral Assessment

A Logitech HD Webcam C922 Pro Stream camera (Logitech, Lausanne, Switzerland) was used to telerecord zebrafish behavior. The recorded videos were examined using ANY-maze^®^ software version 6.3 (Stoelting Co., Wood Dale, IL, USA).

#### 4.7.1. Y-Maze Test

The zebrafish response to novelty was assessed using the Y-maze task [61]. The spatial position in the Y-maze test served as an indicator of memory. Zebrafish were individually trained in a Y-maze glass aquarium (3 L) with three arms (25 × 8 × 15 cm) which were randomly assigned as the “start” arm (always open), the “novel” arm (open during the test trial), and the permanently open arm. During the first training session (5 min), the fish were individually placed in the start arm, and the novel arm was closed. After one hour, the second training session, lasting 5 min, commenced, and the fish were once again positioned in the start arm, but this time, the novel arm was opened. The total distance traveled (m), time spent in the novel arm (% of total arm time), and turn angle (°) were the behavioral parameters investigated in this test. The recorded videos were evaluated using ANY-maze^®^ software (Stoelting Co., Wood Dale, IL, USA).

#### 4.7.2. NOR Test

The NOR test is usually used to assess behavioral memory ability in zebrafish. Zebrafish were briefly subjected to a 5 min acclimatization period in a new tank (30 × 30 × 30 cm filled with 6 cm of water) for three days consecutively with no objects present [61,62,63]. During the training phase (fourth day), zebrafish were introduced to two similar objects for 10 min. One hour after the start of the training stage, one of two similar objects (familiar objects (FOs)) was arbitrarily substituted with a novel object (NO) for 10 min (testing stage). The percentages were determined according to the following: [time of exploration of NO/time of exploration of FO + time of exploration of NO × 100]. The behavior was controlled and analyzed using ANY-maze^®^ software (Stoelting Co., Wood Dale, IL, USA).

### 4.8. Biochemical Analysis

The collected brain tissues were smoothly homogenized (1:10 ratio, w/v) in cold 0.1 M potassium phosphate buffer (pH 7.4) containing 1.15% KCl. The homogenization was carried out for 1 min at 1000 rpm using a Mikro-Dismembrator U mill (Sartorius, New York, NY, USA) equipped with 3 mm diameter magnetic balls (Sartorius Stedim Biotech GmbH, Goettingen, Germany). The resulting homogenate was then subjected to centrifugation for 15 min at 14,000 rpm. The supernatant was collected for further analysis. This included the measurement of the total soluble protein content and the evaluation of the activities of specific enzymes such as acetylcholinesterase (AChE), superoxide dismutase (SOD), catalase (CAT), glutathione peroxidase (GPX); glutathione (GSH); malondialdehyde (MDA) and carbonylated proteins. All procedures were carried out in conformity with the applicable rules and regulations as mentioned in previous studies [60].

### 4.9. Statistical Analysis

The data were presented as means ± standard error of the mean (SEM) and were statistically analyzed using one-way analysis of variance (ANOVA), followed by Tukey’s post hoc multiple comparison tests. The data were visualized using GraphPad Prism v8.3 software (La Jolla, CA, USA). A statistically significant difference was considered at *p* < 0.05.

## 5. Conclusions

The results of this study suggest that the administration of *Tribulus terrestris* ethanolic leaf extract ameliorates cognitive dysfunction and memory impairment as measured via the Y-maze and NOR behavioral tasks. Moreover, Tt treatment reversed SCOP-induced oxidative stress by increasing antioxidant enzyme activities and decreasing the levels of carbonylated proteins and MDA. Our results also indicate a significant inhibition in AChE activity post-treatment, which has been a reliable approach in the management of neurodegenerative diseases, and this inhibition was correspondingly supported by the in-silico data.

These findings highlight the memory-enhancing effects of the Tt extracts and their potential as promising candidates for the management of AD.

## Figures and Tables

**Table 1:** UPLC-ESI/MS peas designation of metabolites in *Tribulus terrestris* (ELE).

No.	Formula	Transformations	Error (ppm)	[M−H]^−1^ Experimental	[M−H]^−1^ Theoretical	Identification
1	C_9_H_10_O_7_	Hydration, Oxidation	−0.03814	180.04225	179.03498	Caffeic acid
2	C_9_H_8_O_3_	Hydration, Oxidation	−0.02587	198.0402	199.0606	Hydroxycinnamic acid
3	C_21_H_20_O_11_	Reduction	−0.38036	196.05823	195.05095	Cynarosie
4	C_15_H_10_O_6_	Nitro Reduction	−0.21529	285.0409	286.24	Kaempferol
5	C_15_H_10_O_6_	Nitro Reduction	0.28338	285.04120	286.24	Luteoline
6	C_21_H_20_O_11_	Reduction	0.17143	301.0353	302.24	Quercetin
7	C_15_H_14_O_7_	Hydration, Oxidation	0.45039	356.07451	306.27	Epigallocatechin
8	C_27_H_42_O_4_	Reduction	−0.38036	255.08642	430.30764	Hecogenine
9	C_21_H_20_O_10_	Reduction	0.18654	435.5	432.4	Apigetrin
10	C_33_H_52_O_8_	Desaturation, Nitro Reduction	2.37949	560.22	576.761	Disogluside–Trillin
11	C_27_H_30_O_16_	Hydration	−1.45637	609.1482	610.1084	Rutin
12	C_39_H_62_O_13_	Desaturation, Nitro Reduction	0.95112	738.05805	738.05077	Trillarin
13	C_39_H_62_O_14_	Desaturation, Nitro Reduction	0.3610	755.262	754.901	Terreside B
14	C_45_H_72_O_17_	Reduction	−4.4	915.4550	915.4590	Terreside A
15	C_45_H_12_O_15_	Desaturation, Nitro Reduction	−0.38036	918.4232	915.4590	Terrestrosin C
16	C_51_H_84_O_22_	Oxidation, Nitro Reduction	−0.10032	1047.5413	1049.2	Protodioscin

**Table 2:** Binding energy of selected compounds with AChE enzyme.

	Compounds	Docking Energy Scores in kcal/mol
	Donepezil (Co-crystal ligand)	−14.62
1	Caffeic acid	−11.22
2	Disogluside (Trillin)	−18.60
3	Apigetrin	−18.60
4	Cynaroside	−18.70
5	Terreside B	−19.98
6	Terrestrosin C	−24.11
7	Trillarin	−23.42
8	Protodioscin	−24.58
9	Epigallocatechin	−20.62
10	Rutin	−24.68
11	Hecogenin	−14.71
12	Saponin C	−24.63
13	Quercetin	−15.39
14	Kaempferol	−15.48
15	Luteoline	−14.65

**Table 3:** Interactions of donepezil, terrestrosin C, protodioscin, rutin, and saponin C within the active site of the AChE enzyme.

Ligand	Affinity Energy (kcal/mol)	Interaction	Amino Acid Distance in Angstrom (Å)
Donepezil (Co-crystal ligand)	−14.62	Attractive Charge	Asp74 (5.40)
		^1^ Hydrogen Bond	Phe295 (1.96)
		^2^ Hydrogen Bond	Ser293 (3.06), Tyr72 (3.20)
		π-Cation	Trp86 (4.73), Tyr337 (3.91)
		π-Sigma	Tyr341 (3.59), Trp286 (3.64)
		π–π Stacked	Trp86 (4.46), Trp86 (3.89, 3.82,5.11), Tyr341 (5.05)
Terrestrosin C	−24.11	^2^ Hydrogen Bond	Gly120 (2.93, 2.88), Asp74 (2.37), Glu202 (2.27), Asn87 (2.33, 2.34), Tyr124 (2.43), Trp86 (2.00)
		^1^ Hydrogen Bond	Tyr124 (2.13), Ser125 (1.76), Trp86 (2.52, 1.68)
		π-Alkyl	Tyr72 (4.65), Tyr124 (4.26), Trp286 (3.20, 4.21, 4.48), Phe297 (4.98), Phe338 (4.91), Tyr341 (4.59, 3.67)
		π-Sigma	Trp286 (2.63)
Protodioscin	−24.58	^1^ Hydrogen Bond	Tyr72 (2.97), Asp74 (2.82), Tyr124 (2.04), Ser293 (2.15, 2.60), Tyr341 (2.75, 2.81), Trp286 (3.23), Trp86 (3.20), Asn87 (2.80, 2.70), Thr83 (2.57), Asp74 (2.65), Glu202 (2.77)
		^2^ Hydrogen Bond	Thr83 (2.78), Glu292 (2.47)
		π-Sigma	Tyr341 (3.48), Tyr337 (3.00)
		π-Alkyl	Tyr124 (4.94), Trp286 (4.76), Phe297 (5.18, 4.56), Phe338 (5.13, 5.01, 4.31, 5.49), Tyr341 (3.80, 4.53), His447 (4.69, 5.41)
Rutin	−24.68	^1^ Hydrogen Bond	Tyr124 (1.64), Phe295 (2.93), Tyr341 (2.45, 3.04), His447 (2.85), Asp74 (2.71), Thr83 (2.79), Asn87 (2.50), Trp86 (3.12), Glu202 (3.05, 3.22)
		^2^ Hydrogen Bond	Trp86 (2.83), Asn87 (2.74), Ser125 (3.02), Ser203 (3.03), Val294 (2.41), His447 (2.36)
		^3^ Hydrogen Bond	His447 (2.91), Trp286 (3.54)
		π–π Stacked	Trp286 (4.85), Tyr337 (3.44), Tyr341 (3.32, 4.03, 5.61)
		π–π T-shaped	Trp86 (5.92), Tyr124 (5.88), Phe338 (4.80)
Saponin C	−24.63	^1^ Hydrogen Bond	Asp74 (2.50), Tyr133 (2.96), Ser293 (2.70), Tyr72 (2.90), Asn87 (3.23), Trp286 (3.36), Leu289 (2.99), Gly120 (2.88)
		^2^ Hydrogen Bond	Gly121 (2.60, 2.69, 2.16), Glu292 (2.84), Phe338 (2.38, 2.41), His447 (2.98), Gln291 (3.54)
		^3^ Hydrogen Bond	Tyr341 (3.58), Trp86 (3.62, 4.12), Phe338 (3.02, 3.73)
		π-Alkyl	Tyr72 (5.33, 4.87), Trp286 (5.33, 4.63, 5.26, 3.88), His287 (4.67), Phe297 (5.29), Phe338 (5.27), Tyr341 (4.84, 4.88)

^1^ Conventional hydrogen bond. ^2^ Carbon hydrogen bond. ^3^ π-donor hydrogen bond.

## Data Availability

The data presented in this study are available on request from the corresponding author.

## Supplementary Materials

The following supporting information can be downloaded at: https://www.mdpi.com/article/10.3390/ph17020200/s1. Table S1: Binding energy, bond type, amino acids and distance of bonds obtained from the docking calculations of tested flavonoids with AChE enzyme. Table S2: 2D representation of predicted binding mode for our selected compounds with AChE enzyme.
