# Supplementary material for: The Effect of a Tribulus-Based Formulation in Alleviating Cholinergic System Impairment and Scopolamine-Induced Memory Loss in Zebrafish (Danio rerio): Insights from Molecular Docking and In Vitro/In Vivo Approaches
Source: Pharmaceuticals (Basel). 2024 Feb 2;17(2):200. doi: 10.3390/ph17020200 (PMC10891926; doi:10.3390/ph17020200)
Supplement: Supplementary file 1 [file pharmaceuticals-17-00200-s001.zip › pharmaceuticals-2791767-supplementary.pdf]

The Effect of a *Tribulus*-Based Formulation in Alleviating Cholinergic System Impairment and Scopolamine-Induced Memory Loss in Zebrafish (*Danio rerio*): Insights from Molecular Docking and *In Vitro*/*In Vivo* Approaches.

Salwa Bouabdallah [1, 2,*], Ion Brinza [2], Razvan Stefan Boiangiu [2], Mona H. Ibrahim [3], Iasmina Honceriu [2], Amna Al-Maktoum [4], Oana Cioanca [5], Monica Hancianu [5], Amr Amin [6,*] , Mossadok Ben-Attia[1], and Lucian Hritcu [2]

[1] Environmental Biomonitoring Laboratory, Bizerte Faculty of Sciences, Carthage University, 7021 Zarzouna, Bizerte, Tunisia. salwamaster@gmail.com (S.B.)
[2] Department of Biology, Faculty of Biology, Alexandru Ioan Cuza University of Iasi,700506 Iasi.
[3] Department of Pharmaceutical Medicinal Chemistry and Drug Design, Faculty of Pharmacy (Girls), Al-Azha University, Cairo 11884, Egypt.
[4] Biology Department, College of Science, United Arab Emirates University, 15551 Al Ain, United Arab Emirates.
[5] Faculty of Pharmacy, "Grigore T. Popa" University of Medicine and Pharmacy, 16 University Street, 700115 Iasi, Romania)
[6] College of Medicine, University of Sharjah, P.O. Box 27272. Sharjah. a.amin@sharjah.ac.ae (A.A.)

Table S1: Binding energy, bond type, amino acids and distance of bonds obtained from the docking calculations of tested flavonoids with with AChE enzyme

		Docking energy scores in kcal/mol	Bond type	Distance in (Å)	Involved amino acids
	Donepezil (Co-crystal ligand)	-14.62	Attractive Charge	5.40	Asp74
			Conventional Hydrogen Bond	1.96	Phe295
			Carbon Hydrogen Bond	3.06	Ser293
			Carbon Hydrogen Bond	3.20	Tyr72
			Pi-Cation	4.73	Trp86
			Pi-Cation	3.91	Tyr337
			Pi-Sigma	3.59	Tyr341
			Pi-Sigma	3.64	Trp286
			Pi-Pi Stacked	4.46	Trp86
			Pi-Pi Stacked	3.89	Trp86
			Pi-Pi Stacked	5.11	Trp286
			Pi-Pi Stacked	3.82	Trp286
			Pi-Pi Stacked	5.05	Tyr341
1	t-Caffeic acid	-11.22	Conventional Hydrogen Bond	2.69	Arg296
			Carbon Hydrogen Bond	2.54	Val294
			Carbon Hydrogen Bond	2.48	Val294
2	Disogluside (Trillin)	-18.60	Conventional Hydrogen Bond	2.31	Tyr124
			Conventional Hydrogen Bond	2.31	Tyr133
			Conventional Hydrogen Bond	2.84	Trp86
			Conventional Hydrogen Bond	2.73	Gly120
			Conventional Hydrogen Bond	3.23	Tyr133
			Carbon Hydrogen Bond	2.83	Ser125
			Carbon Hydrogen Bond	2.61	Gly126

			Carbon Hydrogen Bond<br>Carbon Hydrogen Bond<br>Pi-Sigma<br>Pi-Lone Pair<br>Alkyl<br>Pi-Alkyl<br>Pi-Alkyl<br>Pi-Alkyl<br>Pi-Alkyl<br>Pi-Alkyl<br>Pi-Alkyl<br>Pi-Alkyl<br>Pi-Alkyl<br>Pi-Alkyl<br>Pi-Alkyl<br>Pi-Alkyl<br>Pi-Alkyl<br>Pi-Alkyl<br>Pi-Alkyl<br>Pi-Alkyl<br>Pi-Alkyl<br>Pi-Alkyl<br>Pi-Alkyl<br>Pi-Alkyl	2.33<br>2.79<br>3.46<br>2.91<br>3.99<br>3.88<br>4.79<br>4.77<br>4.27<br>4.87<br>3.70<br>5.07<br>4.58<br>5.45<br>4.98<br>5.26<br>4.20<br>4.60<br>4.74<br>5.11<br>4.34<br>5.06<br>5.32<br>4.55	Gly126<br>Ser125<br>Tyr341<br>Trp86<br>Val294<br>Trp86<br>Trp86<br>Trp86<br>Trp86<br>Trp86<br>Trp86<br>Phe297<br>Tyr337<br>Tyr337<br>Tyr337<br>Phe338<br>Phe338<br>Phe338<br>Tyr341<br>Tyr341<br>Tyr341<br>Tyr341<br>His447<br>His447
3	Apigetrin	-18.60	Conventional Hydrogen Bond<br>Conventional Hydrogen Bond<br>Carbon Hydrogen Bond<br>Carbon Hydrogen Bond<br>Carbon Hydrogen Bond<br>Carbon Hydrogen Bond<br>Carbon Hydrogen Bond<br>Pi-Donor Hydrogen Bond<br>Pi-Donor Hydrogen Bond<br>Pi-Donor Hydrogen Bond<br>Pi-Pi Stacked<br>Pi-Pi Stacked<br>Pi-Pi Stacked<br>Pi-Pi Stacked<br>Pi-Pi Stacked	1.95<br>2.87<br>3.04<br>2.52<br>2.53<br>2.49<br>3.50<br>3.39<br>3.17<br>3.19<br>4.18<br>3.40<br>5.49<br>3.54<br>3.25	Tyr124<br>Tyr124<br>Gly121<br>Gly121<br>Phe338<br>Phe338<br>Ser125<br>Trp286<br>Phe338<br>Trp86<br>Trp286<br>Trp286<br>Tyr341<br>Tyr341<br>Tyr341
4	Cynaroside	-18.70	Conventional Hydrogen Bond<br>Conventional Hydrogen Bond<br>Conventional Hydrogen Bond<br>Conventional Hydrogen Bond<br>Conventional Hydrogen Bond<br>Carbon Hydrogen Bond<br>Carbon Hydrogen Bond<br>Pi-Pi Stacked<br>Pi-Pi Stacked<br>Pi-Pi Stacked<br>Pi-Pi Stacked<br>Pi-Pi Stacked	2.74<br>2.31<br>2.76<br>1.98<br>1.70<br>2.91<br>2.40<br>3.72<br>3.46<br>5.14<br>3.23<br>3.74	Tyr124<br>Phe295<br>Ser125<br>Asn87<br>Asp74<br>Trp86<br>Ser125<br>Trp286<br>Trp286<br>Trp286<br>Tyr341<br>Tyr341
5	Terreside B	-19.98	Conventional Hydrogen Bond<br>Conventional Hydrogen Bond<br>Conventional Hydrogen Bond<br>Conventional Hydrogen Bond<br>Conventional Hydrogen Bond	2.45<br>2.66<br>2.38<br>2.05<br>1.97	Tyr124<br>Ser293<br>Ser293<br>Tyr133<br>Asp74

			Conventional Hydrogen Bond<br>Conventional Hydrogen Bond<br>Carbon Hydrogen Bond<br>Carbon Hydrogen Bond<br>Carbon Hydrogen Bond<br>Pi-Sigma<br>Pi-Alkyl<br>Pi-Alkyl<br>Pi-Alkyl<br>Pi-Alkyl<br>Pi-Alkyl<br>Pi-Alkyl	2.19<br>2.87<br>2.23<br>2.31<br>2.70<br>2.46<br>5.32<br>4.26<br>4.06<br>5.18<br>4.84<br>5.31	Glu202<br>Tyr124<br>Gly121<br>Gly120<br>Glu202<br>Tyr341<br>Trp286<br>Trp286<br>Trp286<br>His287<br>Phe297<br>A:Tyr341
6	Terrestrosin C	-24.11	Carbon Hydrogen Bond<br>Carbon Hydrogen Bond<br>Carbon Hydrogen Bond<br>Carbon Hydrogen Bond<br>Carbon Hydrogen Bond<br>Carbon Hydrogen Bond<br>Carbon Hydrogen Bond<br>Carbon Hydrogen Bond<br>Conventional Hydrogen Bond<br>Conventional Hydrogen Bond<br>Conventional Hydrogen Bond<br>Conventional Hydrogen Bond<br>Pi-Alkyl<br>Pi-Alkyl<br>Pi-Alkyl<br>Pi-Alkyl<br>Pi-Alkyl<br>Pi-Alkyl<br>Pi-Alkyl<br>Pi-Alkyl<br>Pi-Alkyl<br>Pi-Sigma	2.93<br>2.37<br>2.27<br>2.33<br>2.34<br>2.88<br>2.43<br>2.00<br>2.13<br>1.76<br>2.52<br>1.68<br>4.65<br>4.26<br>3.20<br>4.21<br>4.48<br>4.98<br>4.91<br>4.59<br>3.67<br>2.63	Gly120<br>Asp74<br>Glu202<br>Asn87<br>Asn87<br>Gly120<br>Tyr124<br>Trp86<br>Tyr124<br>Ser125<br>Trp86<br>Trp86<br>Tyr72<br>Tyr124<br>Trp286<br>Trp286<br>Trp286<br>Phe297<br>Phe338<br>Tyr341<br>Tyr341<br>Trp286
7	Trillarin	-23.42	Conventional Hydrogen Bond<br>Conventional Hydrogen Bond<br>Conventional Hydrogen Bond<br>Conventional Hydrogen Bond<br>Conventional Hydrogen Bond<br>Carbon Hydrogen Bond<br>Carbon Hydrogen Bond<br>Carbon Hydrogen Bond<br>Carbon Hydrogen Bond<br>Carbon Hydrogen Bond<br>Pi-Donor Hydrogen Bond<br>Pi-Sigma<br>Pi-Alkyl<br>Pi-Alkyl<br>Pi-Alkyl<br>Pi-Alkyl<br>Pi-Alkyl<br>Pi-Alkyl<br>Pi-Alkyl<br>Pi-Alkyl<br>Pi-Alkyl<br>Pi-Alkyl	2.72<br>2.21<br>2.24<br>2.97<br>2.91<br>2.71<br>2.78<br>2.52<br>2.89<br>3.44<br>2.87<br>3.73<br>5.48<br>5.43<br>4.48<br>4.49<br>4.54<br>5.04<br>5.05<br>4.38<br>5.02<br>4.22	Gly121<br>Gly122<br>Tyr124<br>Ser203<br>Asp74<br>Thr83<br>Trp86<br>Ser203<br>His447<br>His447<br>Tyr337<br>Trp286<br>Tyr72<br>Tyr124<br>Tyr124<br>Trp286<br>Trp286<br>Trp286<br>Trp286<br>Trp286<br>Trp286<br>Trp286

			Pi-Alkyl<br>Pi-Alkyl<br>Pi-Alkyl<br>Pi-Alkyl<br>Pi-Alkyl<br>Pi-Alkyl<br>Pi-Alkyl	4.75<br>5.04<br>4.87<br>4.72<br>5.12<br>3.78<br>4.00	His287<br>His287<br>Phe297<br>Phe338<br>Tyr341<br>Tyr341<br>Tyr341
8	Protodioscin	-24.58	Conventional Hydrogen Bond<br>Conventional Hydrogen Bond<br>Conventional Hydrogen Bond<br>Conventional Hydrogen Bond<br>Conventional Hydrogen Bond<br>Conventional Hydrogen Bond<br>Conventional Hydrogen Bond<br>Conventional Hydrogen Bond<br>Conventional Hydrogen Bond<br>Conventional Hydrogen Bond<br>Conventional Hydrogen Bond<br>Conventional Hydrogen Bond<br>Conventional Hydrogen Bond<br>Conventional Hydrogen Bond<br>Carbon Hydrogen Bond<br>Carbon Hydrogen Bond<br>Pi-Sigma<br>Pi-Sigma<br>Pi-Alkyl<br>Pi-Alkyl<br>Pi-Alkyl<br>Pi-Alkyl<br>Pi-Alkyl<br>Pi-Alkyl<br>Pi-Alkyl<br>Pi-Alkyl<br>Pi-Alkyl<br>Pi-Alkyl<br>Pi-Alkyl<br>Pi-Alkyl	2.97<br>2.82<br>2.04<br>2.15<br>2.60<br>2.75<br>2.81<br>3.23<br>3.20<br>2.80<br>2.57<br>2.70<br>2.65<br>2.77<br>2.78<br>2.47<br>3.48<br>3.00<br>4.94<br>4.76<br>5.18<br>4.56<br>5.13<br>5.01<br>4.31<br>5.49<br>3.80<br>4.53<br>4.69<br>5.41	Tyr72<br>Asp74<br>Tyr124<br>Ser293<br>Ser293<br>Tyr341<br>Tyr341<br>Trp286<br>Trp86<br>Asn87<br>Thr83<br>Asn87<br>Asp74<br>Glu202<br>Thr83<br>Glu292<br>Tyr341<br>Tyr337<br>Tyr124<br>Trp286<br>Phe297<br>Phe297<br>Phe338<br>Phe338<br>Phe338<br>Phe338<br>Tyr341<br>Tyr341<br>His447<br>His447
9	Epigallocatech<br>in	-20.62	Conventional Hydrogen Bond<br>Conventional Hydrogen Bond<br>Conventional Hydrogen Bond<br>Conventional Hydrogen Bond<br>Conventional Hydrogen Bond<br>Conventional Hydrogen Bond<br>Conventional Hydrogen Bond<br>Conventional Hydrogen Bond<br>Carbon Hydrogen Bond<br>Carbon Hydrogen Bond<br>Carbon Hydrogen Bond<br>Carbon Hydrogen Bond<br>Carbon Hydrogen Bond<br>Pi-Anion<br>Pi-Donor Hydrogen Bond<br>Pi-Donor Hydrogen Bond<br>Pi-Pi Stacked<br>Pi-Pi T-shaped<br>Pi-Pi T-shaped	3.10<br>3.33<br>2.73<br>2.76<br>2.89<br>2.67<br>2.62<br>3.10<br>2.48<br>2.52<br>2.48<br>2.12<br>2.97<br>4.34<br>3.47<br>3.55<br>3.91<br>5.34<br>4.94	Ser203<br>His447<br>Glu202<br>Glu202<br>Tyr72<br>Thr83<br>Asn87<br>Tyr124<br>Ser125<br>Ser125<br>Ser203<br>Phe338<br>Gly448<br>Asp74<br>Trp86<br>Tyr341<br>Phe338<br>Trp86<br>Trp86

			Pi-Pi T-shaped	5.00	Tyr337
			Pi-Alkyl	4.35	Trp86
10	Rutin	-24.68	Conventional Hydrogen Bond	1.64	Tyr124
			Conventional Hydrogen Bond	2.93	Phe295
			Conventional Hydrogen Bond	2.45	Tyr341
			Conventional Hydrogen Bond	3.04	Tyr341
			Conventional Hydrogen Bond	2.85	His447
			Conventional Hydrogen Bond	2.71	Asp74
			Conventional Hydrogen Bond	2.79	Thr83
			Conventional Hydrogen Bond	2.50	Asn87
			Conventional Hydrogen Bond	3.12	Trp86
			Conventional Hydrogen Bond	3.05	Glu202
			Conventional Hydrogen Bond	3.22	Glu202
			Carbon Hydrogen Bond	2.83	Trp86
			Carbon Hydrogen Bond	2.74	Asn87
			Carbon Hydrogen Bond	3.02	Ser125
			Carbon Hydrogen Bond	3.03	Ser203
			Carbon Hydrogen Bond	2.41	Val294
			Carbon Hydrogen Bond	2.36	His447
			Pi-Donor Hydrogen Bond	2.91	His447
			Pi-Donor Hydrogen Bond	3.54	Trp286
			Pi-Pi Stacked	4.85	Trp286
			Pi-Pi Stacked	3.44	Tyr337
			Pi-Pi Stacked	3.32	Tyr341
			Pi-Pi Stacked	4.03	Tyr341
			Pi-Pi Stacked	5.61	Tyr341
			Pi-Pi T-shaped	5.92	Trp86
			Pi-Pi T-shaped	5.88	Tyr124
			Pi-Pi T-shaped	4.80	Phe338
11	Hecogenin	-14.71	Conventional Hydrogen Bond	2.15	Tyr124
			Conventional Hydrogen Bond	2.13	Tyr133
			Conventional Hydrogen Bond	2.72	Glu202
			Carbon Hydrogen Bond	3.70	Tyr341
			Alkyl	5.29	Val294
			Pi-Alkyl	4.75	Trp86
			Pi-Alkyl	4.98	Trp86
			Pi-Alkyl	4.87	Trp86
			Pi-Alkyl	4.60	Trp86
			Pi-Alkyl	5.01	Tyr124
			Pi-Alkyl	5.08	Tyr124
			Pi-Alkyl	4.78	Trp286
			Pi-Alkyl	4.77	Trp286
			Pi-Alkyl	5.25	Phe297
			Pi-Alkyl	5.05	Tyr337
			Pi-Alkyl	5.08	Tyr337
			Pi-Alkyl	4.91	Phe338
			Pi-Alkyl	4.59	Phe338
			Pi-Alkyl	4.19	Tyr341
			Pi-Alkyl	5.47	Tyr341
			Pi-Alkyl	4.06	Tyr341
			Pi-Alkyl	4.52	His447
			Pi-Alkyl	5.22	His447
12	Saponin C	-24.63	Conventional Hydrogen Bond	2.50	Asp74
			Conventional Hydrogen Bond	2.96	Tyr133
			Conventional Hydrogen Bond	2.70	Ser293
			Conventional Hydrogen Bond	2.90	Tyr72

			Conventional Hydrogen Bond	3.23	Asn87
			Conventional Hydrogen Bond	3.36	Trp286
			Conventional Hydrogen Bond	2.99	Leu289
			Conventional Hydrogen Bond	2.88	Gly120
			Carbon Hydrogen Bond	2.60	Gly121
			Carbon Hydrogen Bond	2.69	Gly121
			Carbon Hydrogen Bond	2.16	Gly121
			Carbon Hydrogen Bond	2.84	Glu292
			Carbon Hydrogen Bond	2.38	Phe338
			Carbon Hydrogen Bond	2.41	Phe338
			Carbon Hydrogen Bond	2.98	His447
			Carbon Hydrogen Bond	3.54	Gln291
			Pi-Donor Hydrogen Bond	3.58	Tyr341
			Pi-Donor Hydrogen Bond	3.62	Trp86
			Pi-Donor Hydrogen Bond	3.02	Phe338
			Pi-Donor Hydrogen Bond	3.73	Phe338
			Pi-Donor Hydrogen Bond	4.12	Trp86
			Pi-Alkyl	5.33	Tyr72
			Pi-Alkyl	4.87	Tyr72
			Pi-Alkyl	5.33	Trp286
			Pi-Alkyl	4.63	Trp286
			Pi-Alkyl	5.26	Trp286
			Pi-Alkyl	3.88	Trp286
			Pi-Alkyl	4.67	His287
			Pi-Alkyl	5.29	Phe297
			Pi-Alkyl	5.27	Phe338
			Pi-Alkyl	4.84	Tyr341
			Pi-Alkyl	4.88	Tyr341
13	Quercetin	-15.39	Conventional Hydrogen Bond	2.66	Gly120
			Conventional Hydrogen Bond	2.53	Tyr133
			Conventional Hydrogen Bond	3.07	Ser203
			Conventional Hydrogen Bond	3.08	Tyr341
			Conventional Hydrogen Bond	2.75	Asn87
			Conventional Hydrogen Bond	3.10	Thr83
			Conventional Hydrogen Bond	2.96	Gly120
			Conventional Hydrogen Bond	2.86	Tyr133
			Conventional Hydrogen Bond	2.66	Glu202
			Carbon Hydrogen Bond	2.47	Thr83
			Carbon Hydrogen Bond	2.53	Trp86
			Carbon Hydrogen Bond	2.93	Gly120
			Pi-Donor Hydrogen Bond	3.79	Tyr337
			Pi-Pi Stacked	4.59	Trp86
			Amide-Pi Stacked	3.95	Gly120;Gly121
14	Kaempferol	-15.48	Carbon Hydrogen Bond	3.00	Gly121
			Carbon Hydrogen Bond	2.87	Gly448
			Pi-Donor Hydrogen Bond	3.48	Trp86
			Pi-Donor Hydrogen Bond	3.44	Tyr337
			Pi-Donor Hydrogen Bond	3.76	Phe338
			Pi-Pi Stacked	4.41	Trp86
			Pi-Pi Stacked	4.31	Trp86
			Pi-Pi Stacked	4.22	Phe338
			Pi-Pi Stacked	5.81	Phe338
15	Luteoline	-14.65	Conventional Hydrogen Bond	3.06	Gly121
			Conventional Hydrogen Bond	2.90	Gly121
			Conventional Hydrogen Bond	2.54	Gly122
			Conventional Hydrogen Bond	2.65	Phe295

			Conventional Hydrogen Bond<br>Conventional Hydrogen Bond<br>Carbon Hydrogen Bond<br>Carbon Hydrogen Bond<br>Carbon Hydrogen Bond<br>Pi-Donor Hydrogen Bond<br>Pi-Donor Hydrogen Bond<br>Pi-Pi Stacked<br>Pi-Pi T-shaped<br>Pi-Pi T-shaped	3.10<br>2.90<br>2.75<br>3.07<br>2.89<br>3.67<br>3.48<br>4.53<br>5.24<br>4.83	His447<br>Ser203<br>Gly121<br>Gly121<br>Val294<br>TRP86<br>TRP86<br>Phe338<br>Tyr337<br>His447

<table>
<tr><td colspan="4">Table S2: 2D representation of predicted binding mode for our selected compounds with AChE enzyme</td></tr>
<tr><td>Cp.</td><td>2D Image</td><td>Cp.</td><td>2D Image</td></tr>
<tr><td>Donepezil (Co-crystal ligand)</td><td>
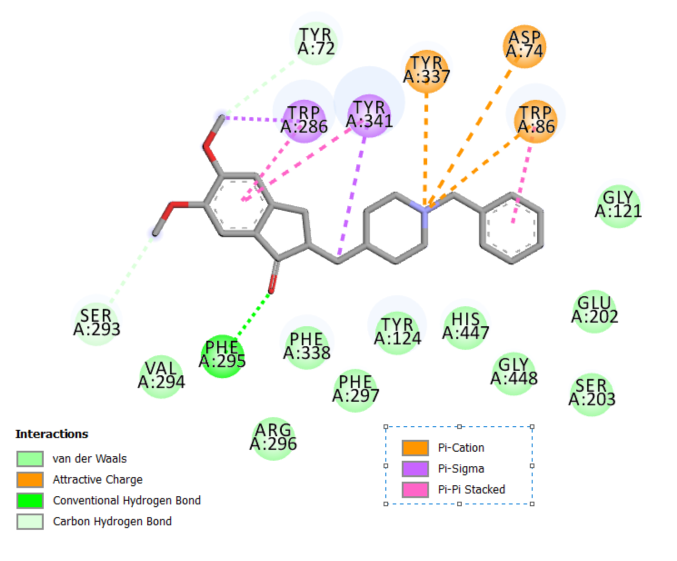

</td><td>t-Caffeic acid</td><td>
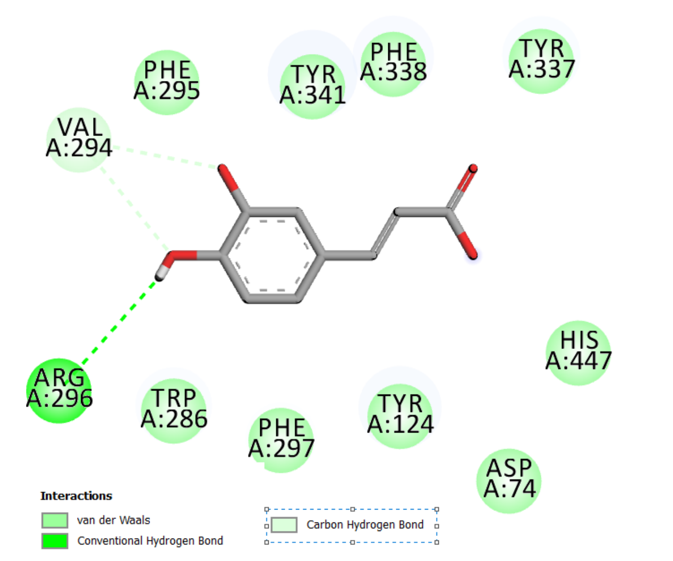

</td></tr>
<tr><td>Disogluside (Trillin)</td><td>
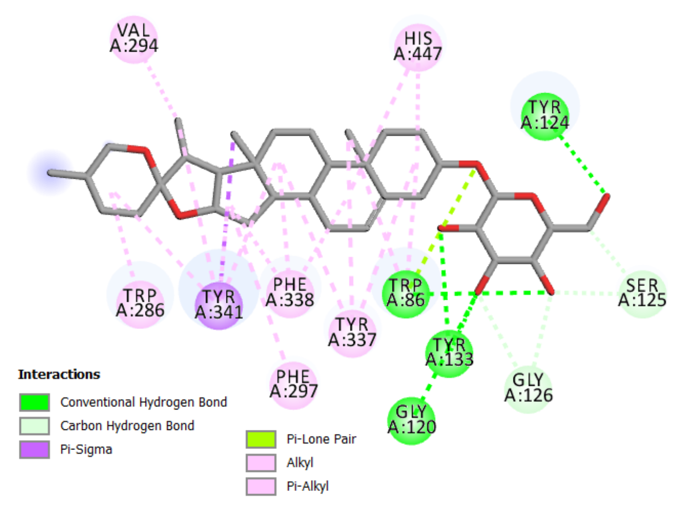

</td><td>Apigetrin</td><td>
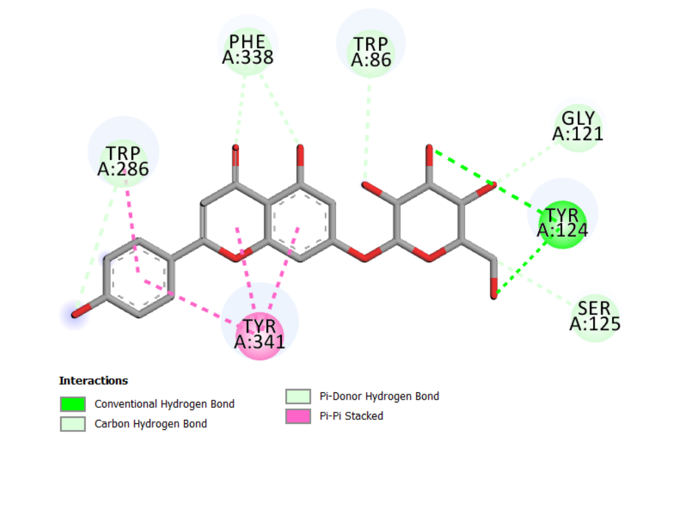

</td></tr>
</table>

Cynaroside	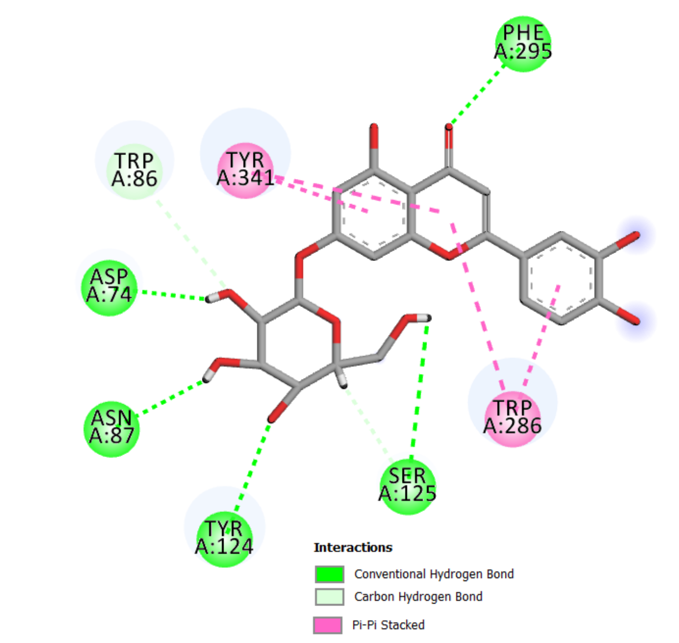	Terreside B	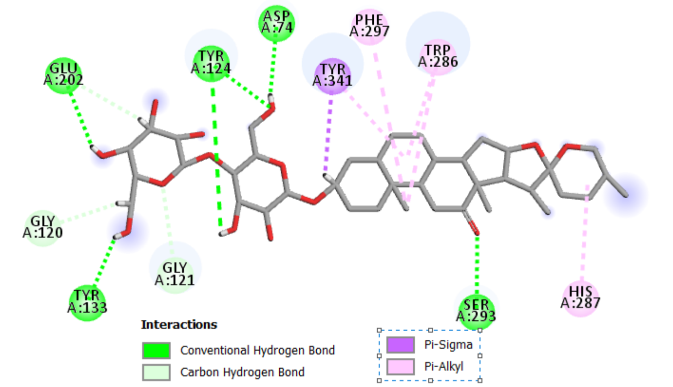
Terrestrosin C	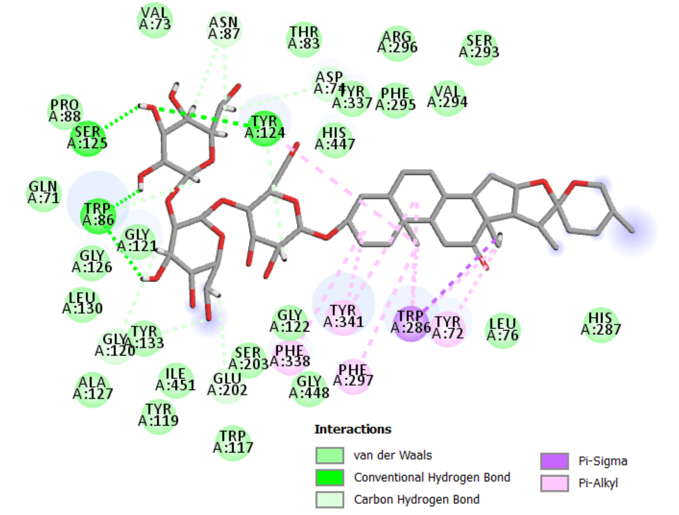	Trillarin	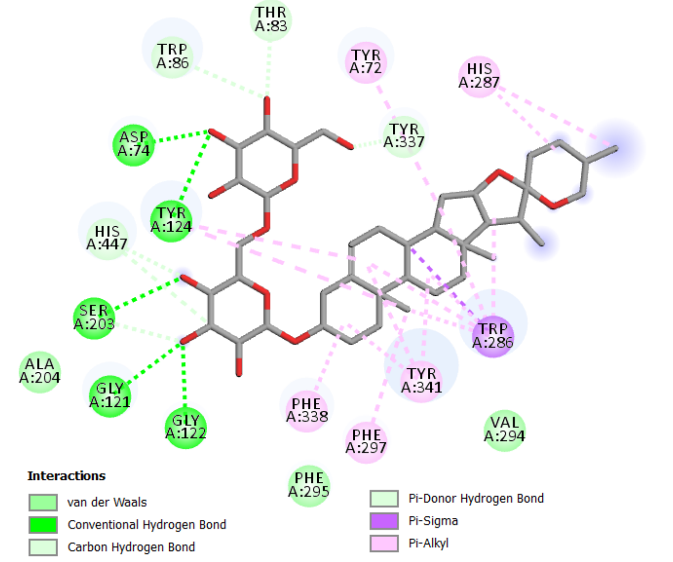

Protodioscin

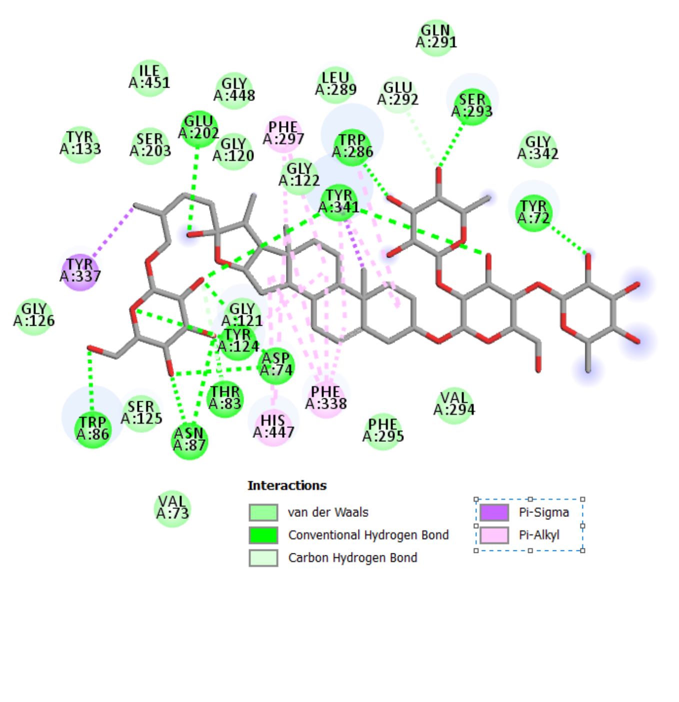

Epigallocatechin

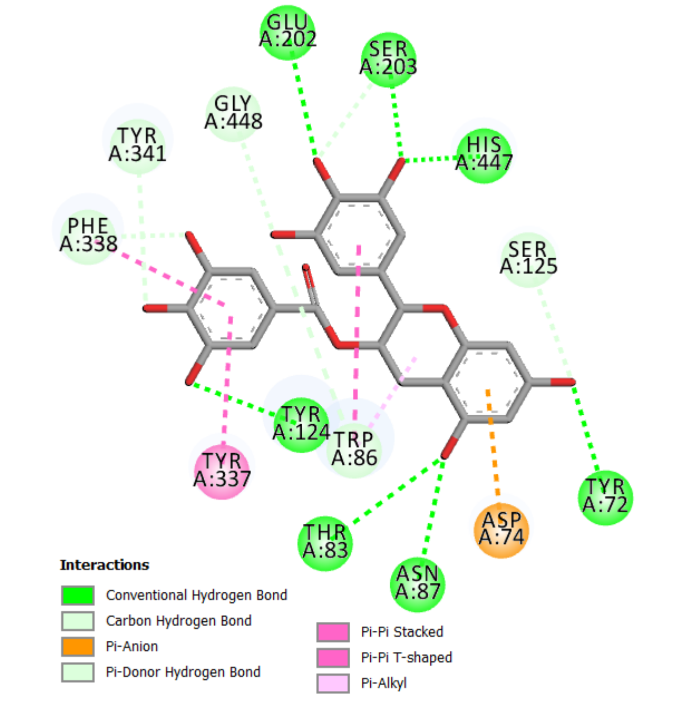

Rutin

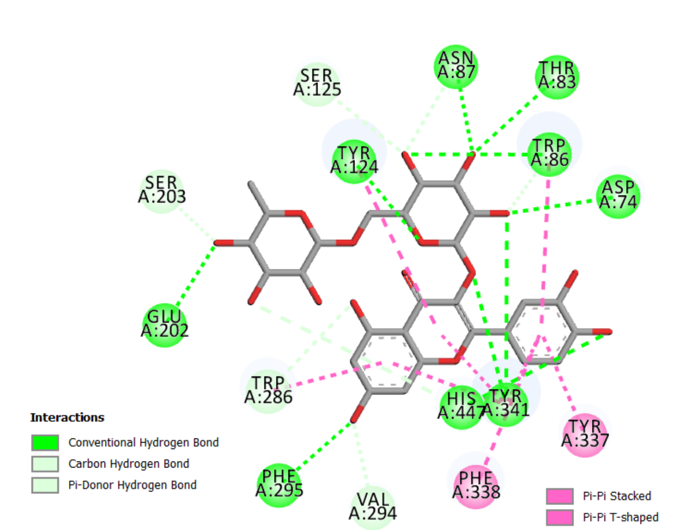

Hecogenin

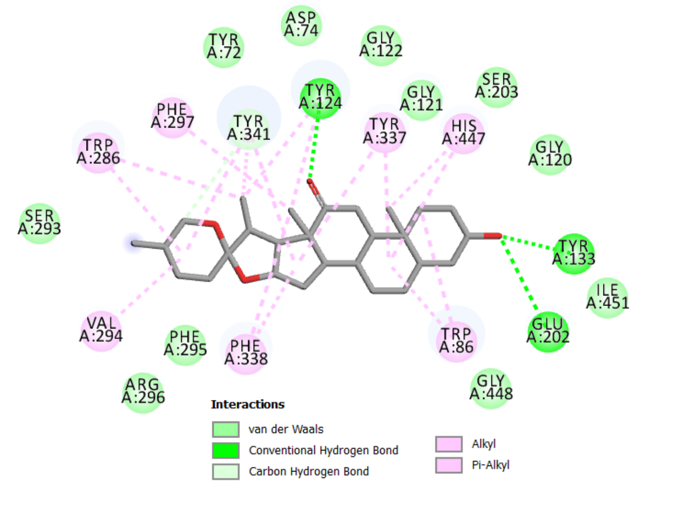

Saponin C	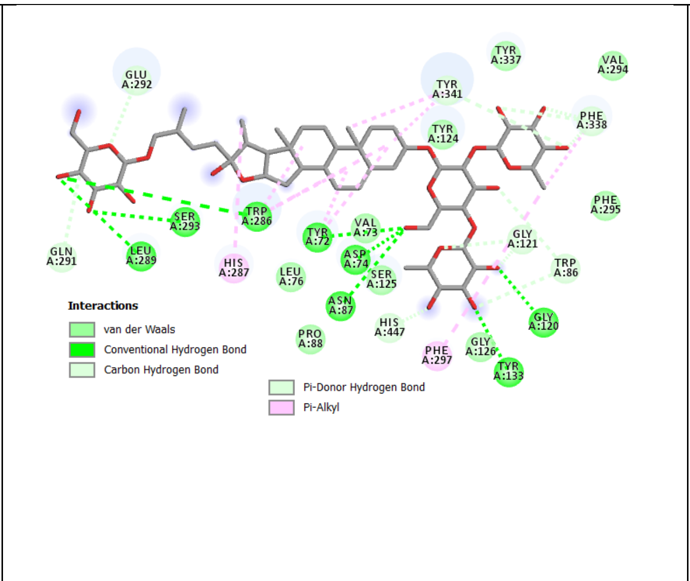	Quercetin	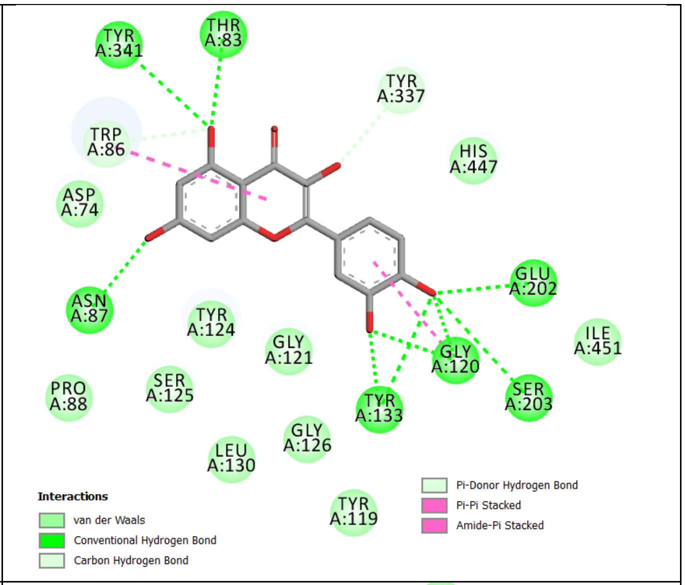
Kaempferol	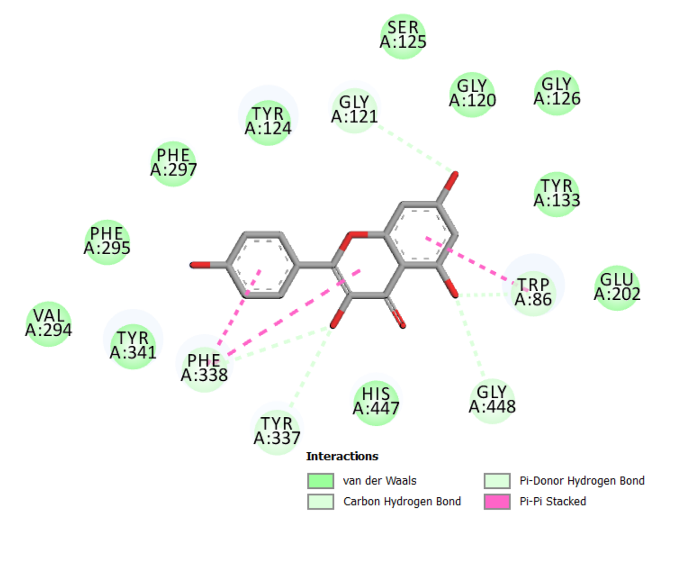	Luteoline	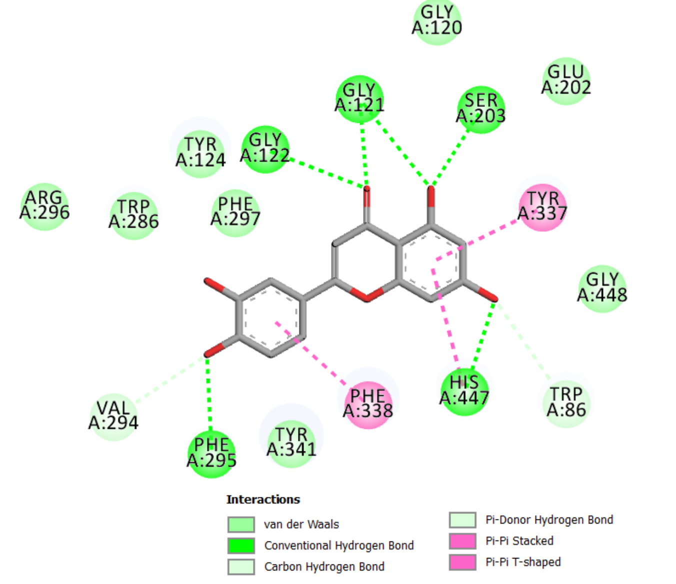
